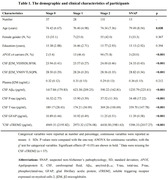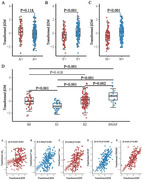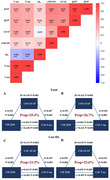# Cerebrospinal Fluid GFAP Mediates the Association of β2‐microglobulin with Tau Pathology

**DOI:** 10.1002/alz.088760

**Published:** 2025-01-03

**Authors:** Ze‐Hu Sheng, Lan‐Yang Wang, Xiao‐Qin Wang, Fan Guo, Yang Lü

**Affiliations:** ^1^ Department of Geriatrics, The First Affiliated Hospital of Chongqing Medical University, Chongqing Medical University, Chongqing, Chongqing China; ^2^ Department of Neurology, Qingdao Municipal Hospital, Nanjing medical university, Qingdao China; ^3^ Department of Geriatrics, The First Affiliated Hospital of Chongqing Medical University, Chongqing Medical University, Chongqing China; ^4^ Department of Neurology, Qingdao Municipal Hospital, Qingdao university, Qingdao, Shandong China; ^5^ The First Affiliated Hospital of Chongqing Medical University, Chongqing, Chongqing China

## Abstract

**Background:**

Recent studies have demonstrated that β2 microglobulin is an important factor in β‐amyloid (Aβ) neurotoxicity and a potential target for the treatment of AD. Although β2 microglobulin, soluble triggering receptor expressed on myeloid cells 2 (sTREM2) and Glial fbrillary acidic protein (GFAP) are involved in the neuroinflammatory response to promote the development of AD, their relationship in AD pathology remains to be studied.

**Method:**

A total of a11 participants with cerebrospinal fuid (CSF) and Plasma β2 microglobulin, CSF sTREM2, GFAP, and AD biomarkers(Aβ_42_; phosphorylated‐tau, P‐tau; and total tau, T‐tau) were included from the Alzheimer’s disease Neuroimaging Initiative (ADNI). We assessed the associations of CSF/Plasma β2 microglobulin with sTREM2/GFAP and AD biomarkers at baseline and in longitudinal study. The mediation models were used to explore the potential mechanism of how β2 microglobulin afects AD pathology.

**Result:**

We found that T+ and N+ groups had higher CSF β2M levels (both p < 0.001), but there was no difference between A+ and A‐ groups. Meanwhile, Since stage 1, CSF β2M levels gradually increased in stage 2 and SNAP. We also found the elevated CSF β2M level was associated with the higher levels of Aβ42 (β = 0.230, p < 0.001), P‐tau (β = 0.564, p < 0.001) and T‐tau (β = 0.603, p < 0.001). There were also positive association between baseline CSF β2M and levels of GFAP (β = 0.552, p < 0.001) and sTREM2 (β = 0.641, p < 0.001). Nevertheless, plasma β2M was not cross‐sectionally correlated with CSF AD core biomarkers, GFAP and sTREM2. Furthermore, only higher CSF β2M was longitudinally associated with slower increase of T‐tau (β = ‐0.025, p = 0.025). Results of mediation analyses showed that the association of CSF β2M with CSF P‐tau (mediating ratio = 25.4%, p < 0.001) and T‐tau (mediating ratio = 26.7%, p < 0.001) was partially mediated by CSF GFAP in total participants. In addition, the mediation effects of sTREM2 were not significant.

**Conclusion:**

Our findings provide evidence to suggest that CSF β2 microglobulin is associated with tau pathology and mediated by CSF GFAP.